# Supplementary material for: Edge magnetism in colloidal MoS2 triangular nanoflakes
Source: RSC Adv. 2026 Jan 9;16(3):2333–41. doi: 10.1039/d5ra08271d (PMC12784245; doi:10.1039/d5ra08271d)
Supplement: RA-016-D5RA08271D-s001 [file RA-016-D5RA08271D-s001.pdf]

Supplementary Information (SI) for RSC Advances.
This journal is © The Royal Society of Chemistry 2025

# SUPPORTING INFORMATION
# Edge Magnetism in Colloidal $MoS_2$ Triangular Nanoflakes

Surender Kumar,[1,*] Stefan Velja,[1] Muhammad Sufyan Ramzan,[1] and Caterina Cocchi[1,†]
[1] *Institut für Festkörpertheorie und -Optik, Friedrich-Schiller-Universität Jena, 07743 Jena, Germany*

## I. STRUCTURAL PROPERTIES

### A. Nearest-Neighbor Bond Distances

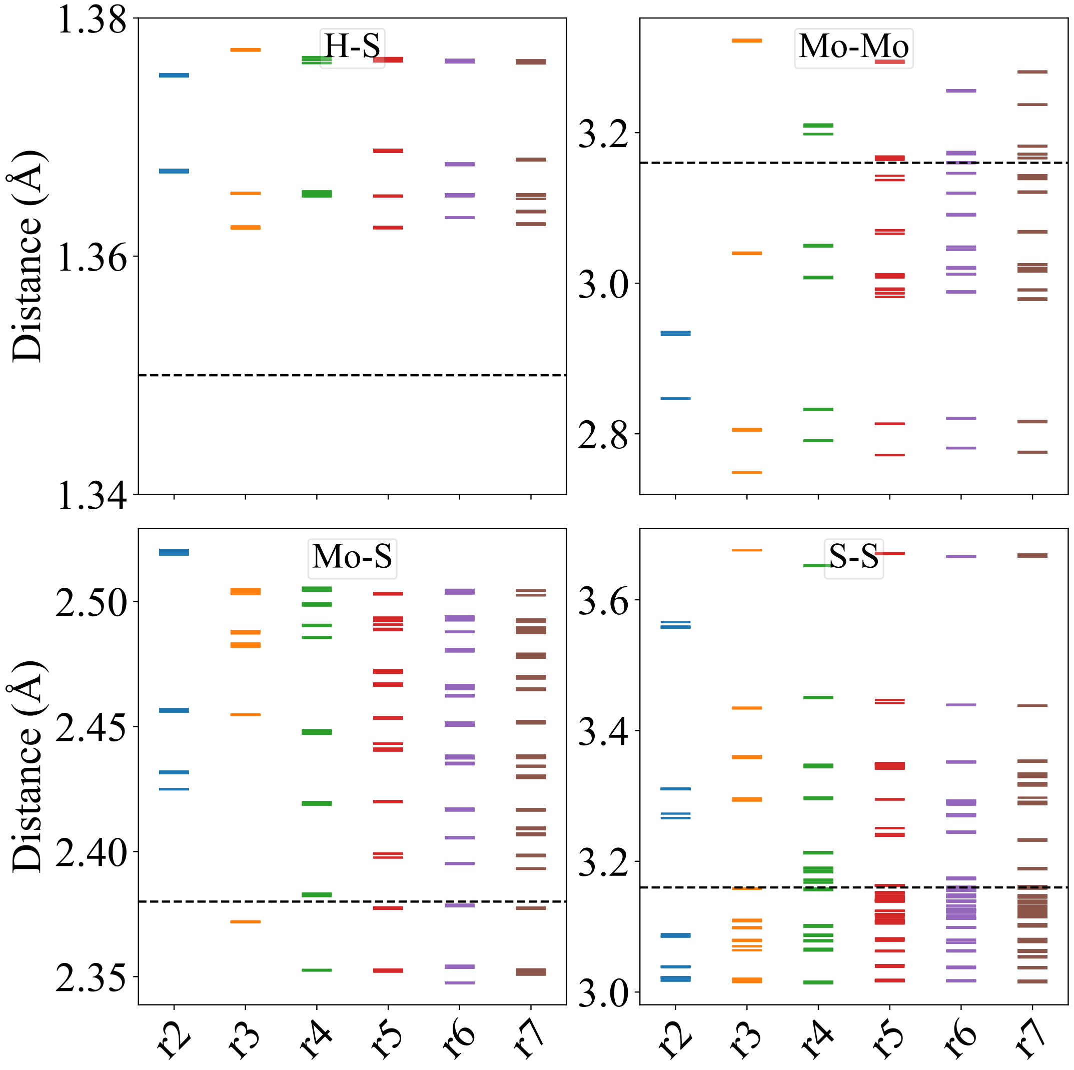


Fig. S1: Nearest-neighbor distances for different atomic pairs (Mo–Mo, Mo–S, S–S, and S–H) in hydrogen-passivated triangular $MoS_2$ nanoflakes after full structural relaxation. Dashed line indicates experimental values.

* surendermohinder@gmail.com
† caterina.cocchi@uni-jena.de

## II. MAGNETIC PROPERTIES

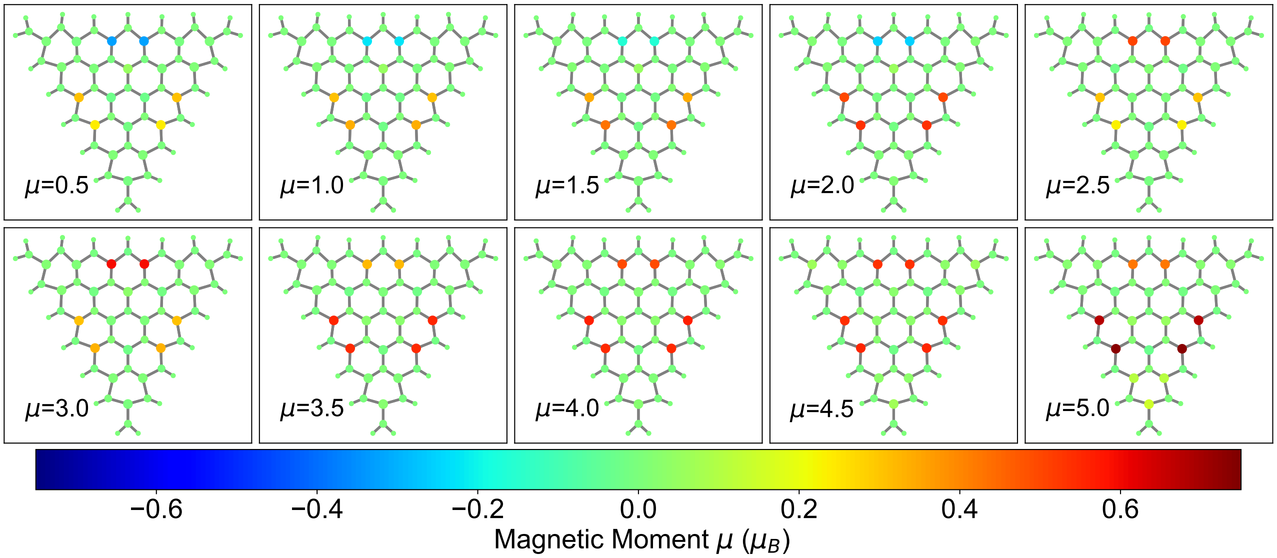


Fig. S2: Spatial distribution of the local magnetic moments in the $r_5$ nanoflake at varying total magnetization (in $\mu_B$) indicated in each panel. The color scale for the atoms represents the magnitude and orientation of the local spins.

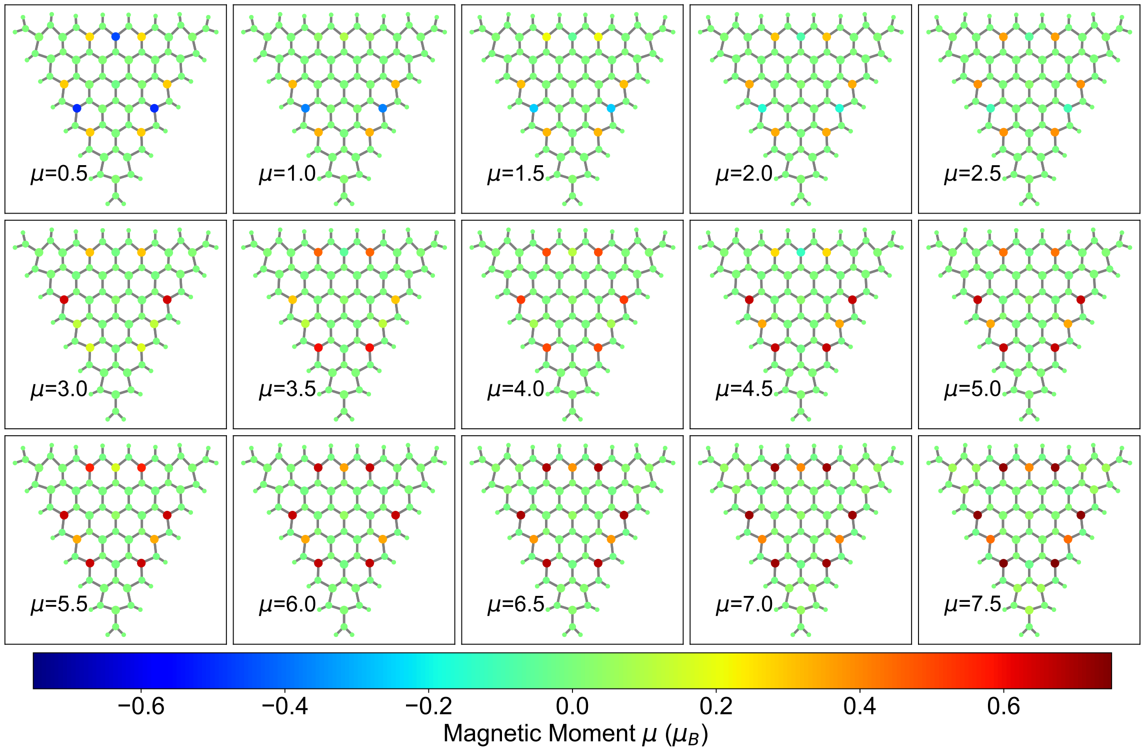


Fig. S3: Spatial distribution of the local magnetic moments in the $r_6$ nanoflake at varying total magnetization (in $\mu_B$) indicated in each panel. The color scale for the atoms represents the magnitude and orientation of the local spins.

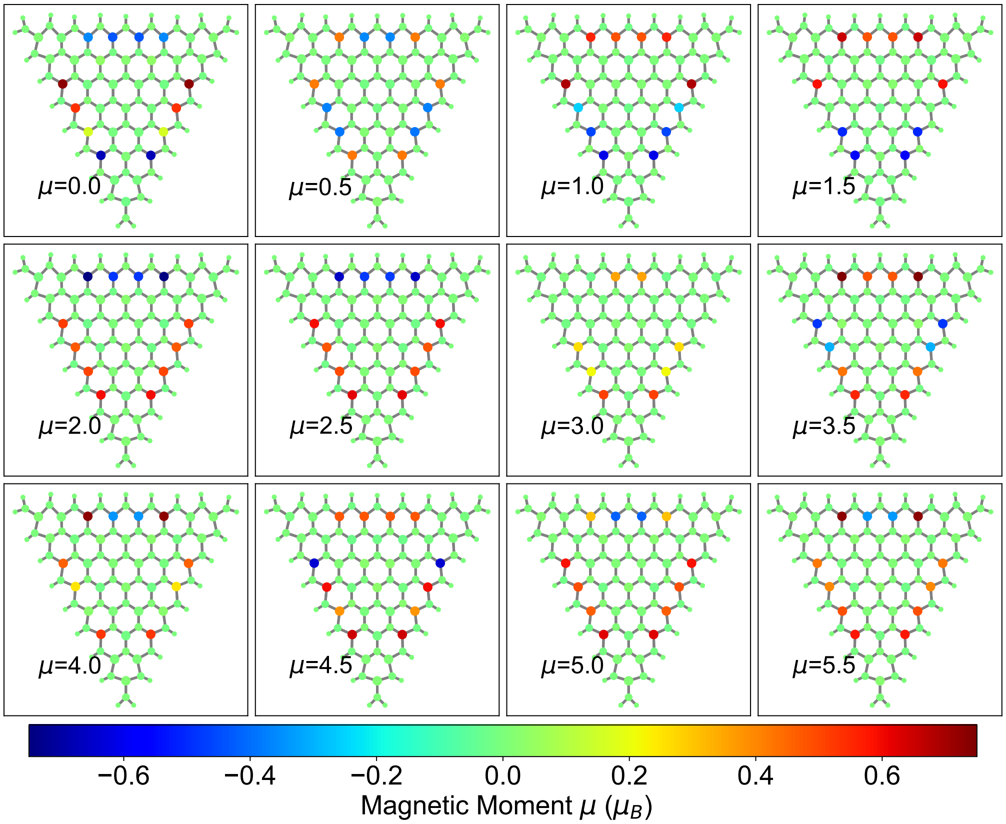


Fig. S4: Spatial distribution of the local magnetic moments in the $r_7$ nanoflake at varying total magnetization (in $\mu_B$) indicated in each panel. The color scale for the atoms represents the magnitude and orientation of the local spins.

## III. EFFECT OF HYDROGEN PASSIVATION

In Figure S5, we show the spatial distribution of local magnetic moments in all considered nanoflakes H-passivation. Opposite to their H-passivated counterparts discussed in the main text, non-passivated flakes exhibit a net magnetic moment even at the smallest $r_2$ size. The bare edges fundamentally change the source of magnetism, forcing both Mo and S atoms to contribute to the local magnetic moments. The magnetism of the S-atoms primarily originates from the unsaturated *p*-orbitals of the S-edge atoms.

The spin distribution is highly size-dependent and increasingly disordered with increasing flake size. The $r_2$ nanoflake is magnetic due to an unpaired spin localized on a single Mo atom, while the $r_3$ flake shows spin polarization across several edge Mo atoms. In the larger flakes ($r_4$ and $r_5$), the spin pattern becomes more complex, with different spin orientations mainly localized on S atoms and only small, residual magnetic moments on Mo atoms. Interestingly, the $r_6$ flake shows a distinct pattern, where large local magnetic moments appear specifically on the S atoms located at the corners, a feature partially reminiscent of the smaller structures. In the largest nanoflake studied, $r_7$, the overall local magnetic moments are significantly reduced, indicating a size-dependent suppression of edge magnetism in this bare-edge configuration.

Overall, these results confirm that H-passivation is essential for stabilizing and regularizing edge magnetism, channeling it into a controlled, confined, and Mo-dominated spin activity. Its absence results in a spatially random, complex spin distribution highly sensitive to the size of the nanoflake, and dominated by the more reactive sulfur edge states, highlighting the critical role of chemical passivation in designing controllable TMD spintronic platforms.

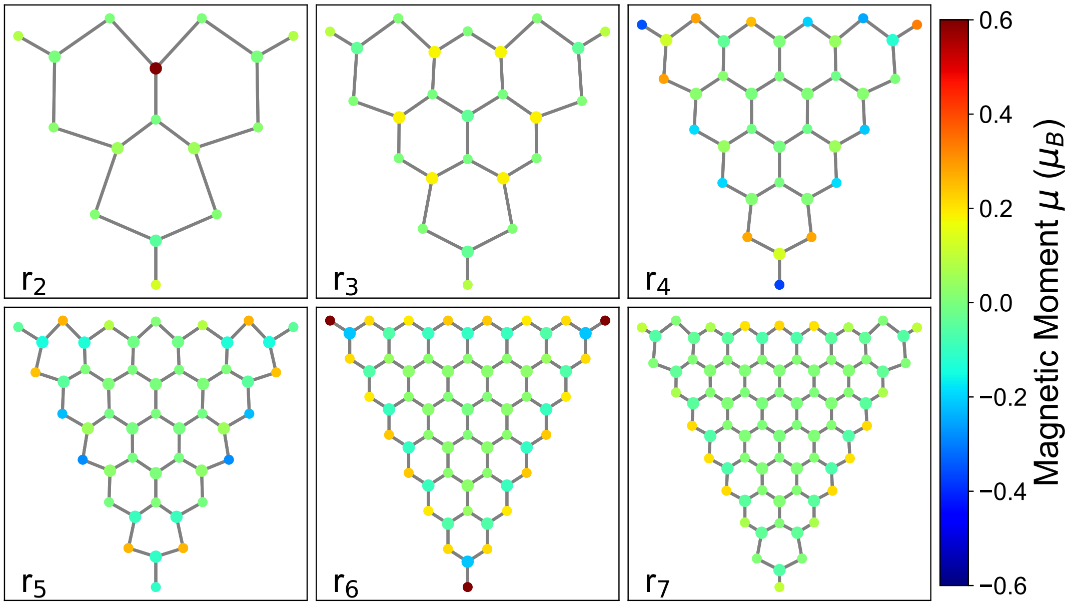


Fig. S5: Spatial distribution of the local magnetic moments in the unpassivated $MoS_2$ nanoflakes. The color scale for the atoms represents the magnitude and orientation of the local spins.

## IV. STRUCTURALLY MODIFIED TRIANGULAR NANOFLAKES

Colloidal $MoS_2$ nanoflakes synthesized in solution are often characterized by irregular geometries, which may slightly depart from the equilateral structures considered in this study. To test the robustness of the discussed physics with respect to the nanoflake shape, we consider an additional set of model systems characterized by smaller structural deviations from the ideal equilateral geometry, obtained by removing one, two, or three Mo atoms from the corners (Figure S7).

The considered irregular structures of all sizes remain magnetic with spin patterns very similar to those found in the equilateral configuration. Only minor additional magnetic moments appear on the atoms situated near the removed Mo, indicating a localized perturbation of the magnetic landscape. These findings demonstrate that the atom-selective edge magnetism is remarkably robust against moderate geometric distortions.

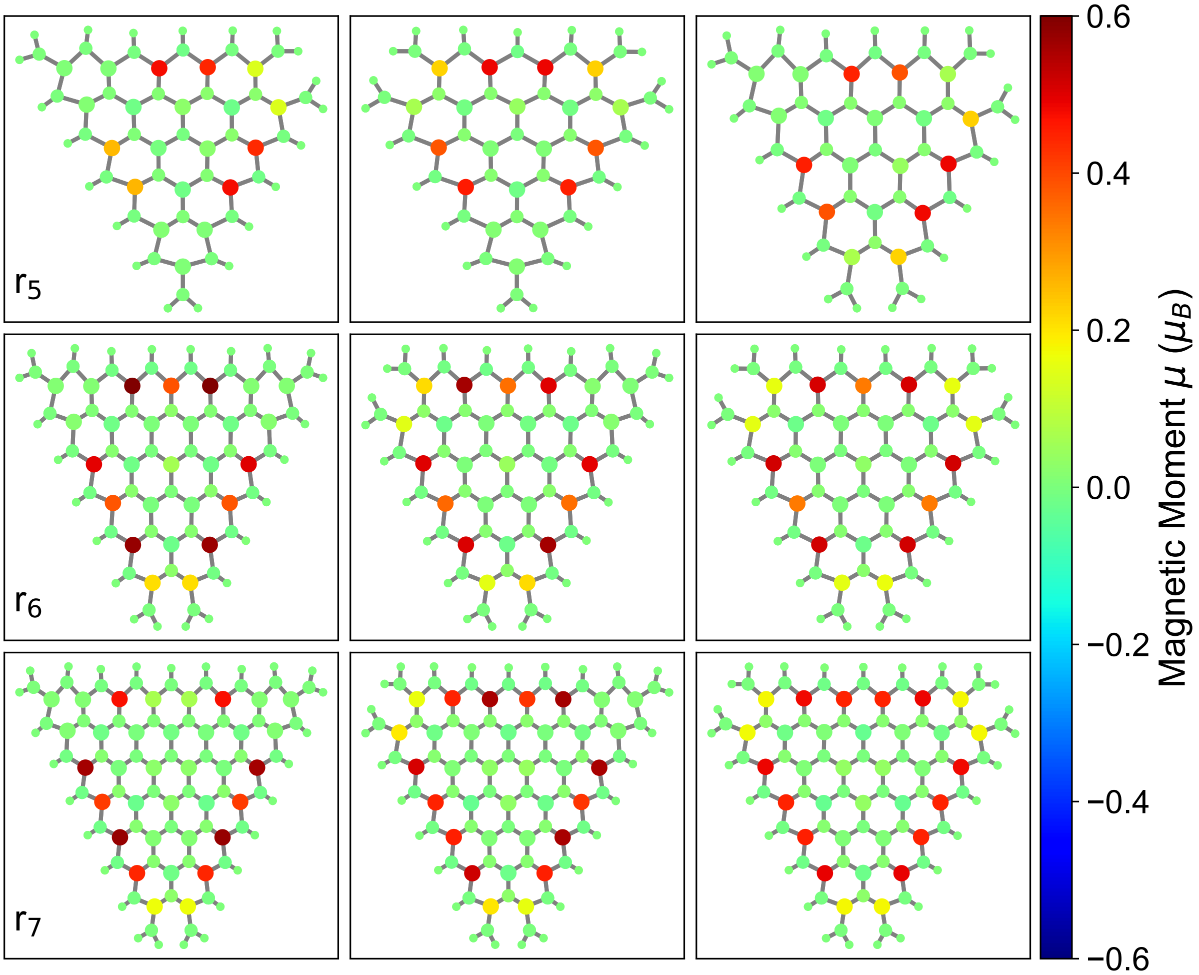


Fig. S6: Spatial distribution of the local magnetic moments in the $r_5$, $r_6$, and $r_7$ nanoflakes with geometries deviating from the ideal equilateral triangular shape. The color scale for the atoms represents the magnitude and orientation of the local spins.

## V. SINGLE PARTICLE ENERGY LEVELS

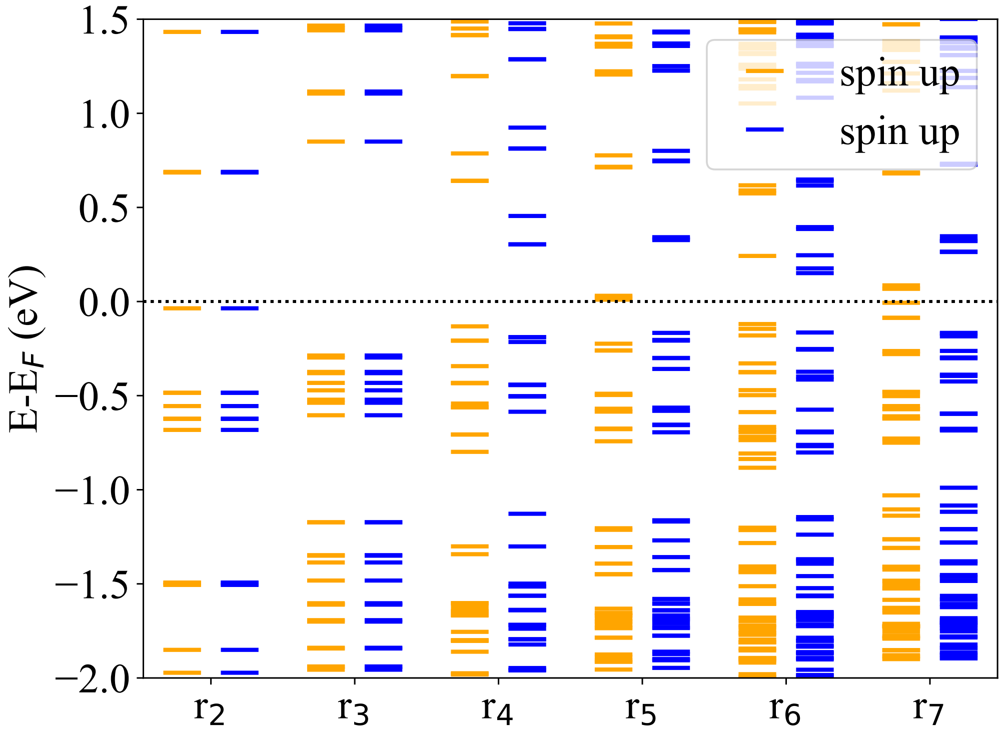


Fig. S7: Single particle energy levels for calculated structure ($r_2$-$r_7$) from main text.
